# Skin transcriptome profiles associated with coat color in sheep

**DOI:** 10.1186/1471-2164-14-389

**Published:** 2013-06-10

**Authors:** Ruiwen Fan, Jianshan Xie, Junming Bai, Haidong Wang, Xue Tian, Rui Bai, Xiaoyun Jia, Lei Yang, Yunfei Song, Muren Herrid, Wenjun Gao, Xiaoyan He, Jianbo Yao, George W Smith, Changsheng Dong

**Affiliations:** 1 College of Animal Science and Veterinary Medicine, Shanxi Agricultural University, Taigu, 030801, China; 2 School of Science and Technology, University of New England, Armidale, NSW, 2351, Australia; 3 Laboratory of Animal Biotechnology and Genomics, Division of Animal and Nutritional Sciences, West Virginia University, Morgantown, WV, 26506, USA; 4 Departments of Animal Science and Physiology, Laboratory of Mammalian Reproductive Biology and Genomics, Michigan State University, East Lansing, MI, 48824, USA

**Keywords:** Sheep skin, Transcriptome, Gene expression, Pigmentation, Melanogenesis

## Abstract

**Background:**

Previous molecular genetic studies of physiology and pigmentation of sheep skin have focused primarily on a limited number of genes and proteins. To identify additional genes that may play important roles in coat color regulation, Illumina sequencing technology was used to catalog global gene expression profiles in skin of sheep with white versus black coat color.

**Results:**

There were 90,006 and 74,533 unigenes assembled from the reads obtained from white and black sheep skin, respectively. Genes encoding for the ribosomal proteins and keratin associated proteins were most highly expressed. A total of 2,235 known genes were differentially expressed in black versus white sheep skin, with 479 genes up-regulated and 1,756 genes down-regulated. A total of 845 novel genes were differentially expressed in black versus white sheep skin, consisting of 107 genes which were up-regulated (including 2 highly expressed genes exclusively expressed in black sheep skin) and 738 genes that were down-regulated. There was also a total of 49 known coat color genes expressed in sheep skin, from which 13 genes showed higher expression in black sheep skin. Many of these up-regulated genes, such as *DCT, MATP, TYR and TYRP1,* are members of the components of melanosomes and their precursor ontology category.

**Conclusion:**

The white and black sheep skin transcriptome profiles obtained provide a valuable resource for future research to understand the network of gene expression controlling skin physiology and melanogenesis in sheep.

## Background

Sheep are the most important fiber producing animals worldwide. Fiber diameter, length and color are key traits contributing to the economic value of sheep and are determined by both genetics [1,2] and environment [3]. Factors that determine coat color in sheep are becoming of increasing interest. White fleece holds greatest economic value due to its ability to be dyed to virtually any color, whereas interest in natural colors is increasing due to the green revolution and consumer preference for natural products.

Coat color genes are good candidates for facilitation of traceability of animal breeds [4]. Coat color is determined by amounts and types of melanin produced and released by melanocytes resident in the skin [5,6]. The genetic basis for coat color is well understood in rodents [7,8], with many common genes also implicated in regulation of coat color in other species, including sheep. For example, *MC1R* and *ASIP* are known to be major regulators of coat color in mice and *MC1R*[9] and *ASIP*[10] loci are functionally linked to undesirable coat color phenotypes in sheep. In addition, tyrosinase-related protein 1 *(TYRP1)* is a strong positional candidate gene for color variation in Soay sheep [11]. Recent studies have combined SNP analysis and gene expression profiling to dissect the basis for the piebald pigmentation phenotype in Merino sheep [12]. Despite considerable knowledge of the genetic regulation of coat color in mice and identification of loci involved in coat color regulation in fiber producing species, the molecular mechanisms, at the level of gene expression, associated with differences in coat color phenotype are not well understood. This information is critical not only to enhanced basic understanding of regulation of melanogenesis, but also to the identification of novel pharmacological and molecular genetics approaches to regulate or select for coat color in fiber producing species.

Transcriptional profiling is a powerful approach for identification of genes globally and functionally expressed in various tissues including skin [13]. Limited information is currently available regarding differences in transcriptome profiles of skin associated with coat color in fiber producing species. To investigate genes that may play important roles in coat color regulation in sheep skin and gain insight into molecular mechanisms responsible for biochemistry of skin and fibers (including pigmentation) in animals producing hair such as sheep and alpaca, we investigated the transcriptome profiles in skin of sheep with black versus white coat color using high throughput RNA deep sequencing. Results provided novel insight into differences in gene expression associated with coat color, including key genes implicated in the melanogenesis pathway.

## Results

### Assembly of unigenes

**Figure 1:**
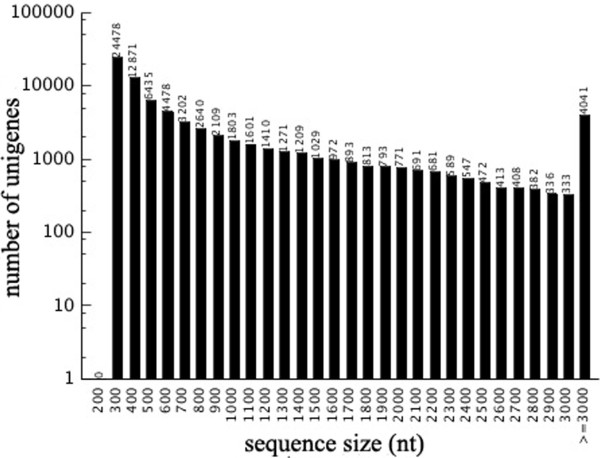
Length distribution and abundance of all unigenes identified in sheep skin.

After the raw reads were filtered, 51,297,002 clean reads with 52.2% GC percentage and 51,655,390 clean reads with 53.7% GC percentage were obtained from white and black sheep skin, respectively. These clean reads were assembled into unigenes, yielding 90,006 and 74,533 unigenes from white and black sheep skin, respectively. There were 2,892 and 2,884 unigenes with sequence size greater than 3,000 nucleotides in white and black sheep skin, respectively. The longest unigene sequenced was more than 9,000 nt in length and the average size of the majority of coding sequence (CDS) identified was 300 nt. There were 1,367 unigenes with more than 3,000 nt of CDS (Figure 1).

### Functional classification of the unigenes

**Figure 2:**
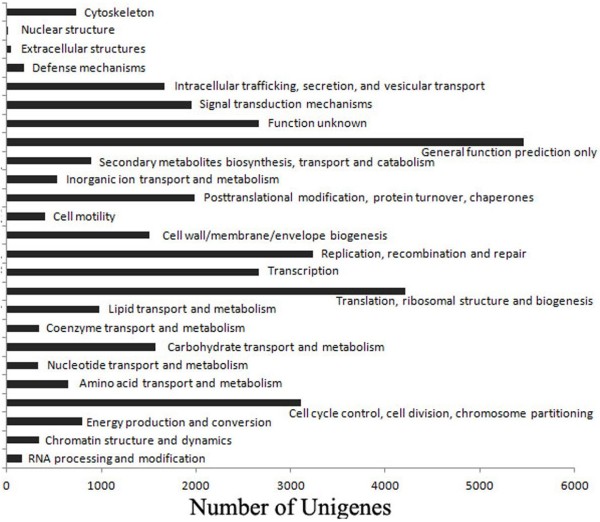
COG functional classification of all unigenes in sheep skin.

**Figure 3:**
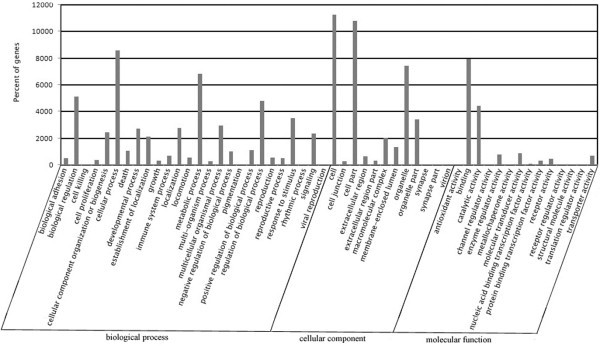
GO functional classification of all unigenes in sheep skin.

BLAST analysis (e-value < 0.00001) of the sheep skin unigenes against the protein and nucleotide databases revealed 37,768 known genes, of which, 36,438 were annotated through COG classification analysis. These genes were grouped into 25 classes based on their putative functions and the largest group of genes was classified into general function only (15%; Figure 2). The known genes were also annotated through GO classification analysis and grouped into 3 categories (biological process, 46.1%; cellular component, 36.1%; molecular function, 16%) based on their putative functions (Figure 3).

### Genes highly expressed in sheep skin

The top 30 genes most highly expressed in sheep skin included genes of the keratin family and ribosomal proteins (Table 1). The most highly expressed gene in sheep skin was 40S ribosomal protein S29. The other highly expressed genes in sheep skin included, NADH dehydrogenase subunit 5 (*NADH5*), cytochrome c oxidase subunit I (*COX1*), NADH-ubiquinone oxidoreductase chain 4 (*ND4*), keratin associated protein 9.2 (*KAP9.2*), keratin 27 (*Krt27*), high-sulfur keratin BIIIB4 protein, hair keratin cysteine rich protein (*Dmpk*), keratin-associated protein 1.4 (*KRTAP1.4*) and keratin-associated protein 3–2 (*KAP3.2*). The list of most highly expressed genes also included 4 unknown genes.

**Table 1:** Top 30 highly expressed genes in sheep skin

**Gene name**	**FPKM (White)**	**FPKM (Black)**
Keratin associated protein 9.2	2584.8	821.2
Keratin 27	2607.2	1067.3
Complete mitochondrial genome	2674.7	3041.1
Unknown (unigene10779_all)	2748.0	3383.1
PREDICTED: ferritin, heavy polypeptide 1	2888.1	2553.0
Ribosomal protein P2-like	2895.4	2725.2
SJCHGC01393 protein	2901.1	3225.6
NADH dehydrogenase subunit 5	2932.5	2388.7
Cystatin-M precursor	2986.9	782. 3
Unknown	3057.6	2405.2
40S ribosomal protein S15	3076.5	2540.7
High-sulfur keratin BIIIB4 protein	3091.8	1541.3
60S ribosomal protein l18a-like	3167.0	3583.3
40S ribosomal protein S8	3400.9	3428.3
40S ribosomal protein S17	3456.8	3098.2
40S ribosomal protein S2	3617.6	3951.0
PREDICTED: 40S ribosomal protein s15a	3621.0	3604.1
High-sulfur keratin BIIIB4 protein	3717.6	2178.6
Hair keratin cysteine rich protein	3729.8	2563.5
Keratin-associated protein 9.2	3753.7	1379.9
Bos taurus ubiquitin C (UBC)	3912.4	5532.3
Keratin-associated protein 1.4	3945.3	2057.2
40S ribosomal protein S11	4019.3	3719.5
PREDICTED: rrna promoter binding protein-like	4062.3	4910.0
Keratin associated protein 9.2	4628.2	1689.3
Cytochrome c oxidase subunit I	5279.6	3998.8
Keratin-associated protein 3-2	6143.7	3911.5
NADH-ubiquinone oxidoreductase chain 4	6684.7	5468.1
Unknown (unigene19263_all)	7622.1	12646.3
PREDICTED: 40S ribosomal protein S29-like	19925.8	9994.6

### Genes encoding transcription factors expressed in sheep skin

In the transcriptome of sheep skin, there was at least 527 genes identified encoding for transcription factors (Additional file 1: Table S1). The highly expressed transcription factor genes in both white and black sheep skin include transcription factor jun-B-like*,* nascent polypeptide-associated complex subunit alpha isoform b, endothelial differentiation-related factor 1 isoform alpha, homeobox protein DLX-3 (*DLX3*), transcription factor AP-1 (*JUN*), cyclic AMP-dependent transcription factor ATF-4 (*ATF4*), and transcription factor GATA3 (*GATA3*) (Table 2). Most of these transcription factors do not show differential expression between white and black skin. However, some less abundantly expressed transcription factors such as *Pbx*, *Tcf4*, *Nr2f1*, *Sox11* and *Sox4*, were differentially expressed in white and black sheep skin. Transcription factors that are known to regulate mRNA expression of coat color genes, such as *MITF* and *CREB/ATF bZIP* transcription factor were also found to be expressed in sheep skin. Interestingly, both genes were expressed approximately 2 times higher in white sheep skin than black sheep skin.

**Table 2:** Highly expressed transcription factors in sheep skin

**Gene name**	**FPKM (White)**	**FPKM (Black)**
Transcription factor jun-B-like	206.6	368.3
Cyclic AMP-dependent transcription factor ATF-4	301.0	340.3
Transcription factor AP-1	125.5	205.5
Endothelial differentiation-related factor 1 isoform alpha	94.0	140.7
homeobox protein DLX-3	132.6	90.1
transcription factor GATA3	61.7	63.2

### Differentially expressed genes in white versus black sheep skin

**Figure 4:**
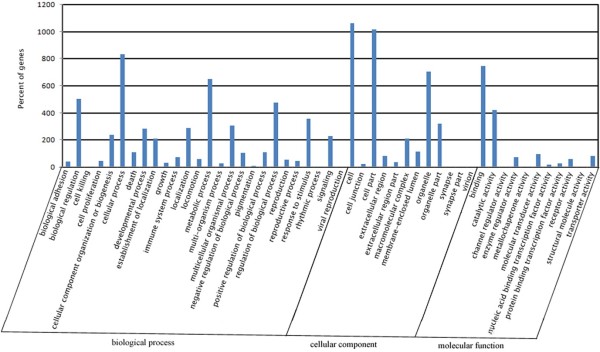
GO functional classification of differentially expressed unigenes in black versus white sheep skin.

Using an algorithm based on a previously described method [14], genes differentially expressed between white and black sheep skin were identified. There were a total of 2,235 known genes identified as differentially expressed in white versus black sheep skin, of which 1,756 were down-regulated (≤ 2 fold) and 479 were up-regulated (≥ 2 fold) in skin from black sheep compared with skin from white sheep (see Additional file 2: Table S2). For the GO analysis, 1,904, 1,784 and 1,787 differentially expressed genes were grouped in cellular component, molecular function and biological process categories, respectively. Most of the differentially expressed genes were classified into two GO categories (cellular process and cell and cell part; Table 3 and Figure 4). The majority of the GO terms including pigmentation do not appear to be significantly enriched in the differentially expressed genes.

**Table 3:** Total number of genes and genes with difference in each GO term

**GO terms**	**Total number of genes**	**Total number of genes with difference**	**Corrected P-value**
Biological adhesion	673	89	1
Biological regulation	8650	1141	1
Cell killing	32	5	1
Cell proliferation	470	76	1
Cellular component organization or biogenesis	3698	515	1
Cellular process	11404	1441	1
Death	678	92	1
Developmental process	3927	549	1
Establishment of localization	3191	416	1
Growth	309	34	1
Immune system process	1278	175	1
Localization	3783	92	1
Locomotion	882	126	1
Metabolic process	7062	944	1
Multi-organism process	963	132	1
Multicellular organismal process	5437	642	1
Negative regulation of biological process	2792	398	1
Pigmentation	69	21	0.40
Positive regulation of biological process	3228	447	1
Regulation of biological process	8093	1078	1
Reproduction	1347	147	1
Reproductive process	1342	147	1
Response to stimulus	6174	753	1
Rhythmic process	184	21	1
Signaling	3997	469	1
Viral reproduction	534	43	1
Cell	13120	1724	7.08 × 10^-8^
Cell junction	679	86	1
Cell part	13110	1724	4.13 × 10^-8^
Extracellular region	1685	256	0.15
Extracellular region part	960	157	0.09
Macromolecular complex	3942	495	1
Membrane-enclosed lumen	3111	420	1
Organelle	9471	1284	6.93 × 10^-5^
Organelle part	6280	845	0.72
Synapse	477	61	1
Synapse part	353	44	1
Antioxidant activity	55	4	
Binding	10938	1519	1.11 × 10^-15^
Catalytic activity	5291	725	1
Channel regulator activity	76	13	1
Enzyme regulator activity	903	123	1
Molecular transducer activity	1898	136	1
Nucleic acid binding transcription factor activity	817	116	1
Protein binding transcription factor activity	495	66	1
Receptor activity	2057	157	1
Receptor regulator activity	35	5	1
Structural molecule activity	792	76	1
Translation regulator activity	27	2	1
Transporter activity	1175	153	1

A total of 845 novel genes were identified as differentially expressed, of which 738 were down-regulated (≤ 2 fold) and 107 were up-regulated (≥ 2fold) in skin from black sheep compared with skin from white sheep. Of the 107 up-regulated genes, 16 genes were exclusively expressed in black sheep skin (including 2 genes highly expressed), and 26 genes were expressed 8 to 256 times higher in black versus white sheep skin (see Additional file 3: Table S3).

**Figure 5:**
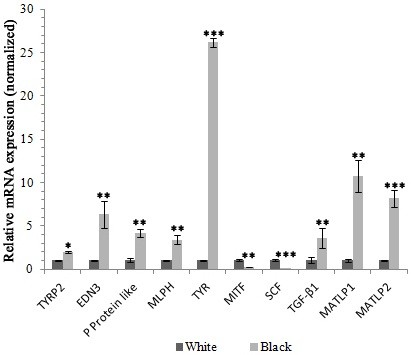
**Real time PCR validation of differentially expressed genes in black versus white sheep skin.** Abundance of target genes was normalized relative to abundance of â-actin gene. Bars in each panel represent the mean ± standard error (n = 3), * P < 0.05; ** P < 0.01; *** P < 0.001.

In order to validate the transcriptome sequencing results, we selected 10 genes at random for real time PCR to determine their relative expression in black and white sheep skin. These genes, identified as differentially expressed in black versus white sheep skin based on transcriptome sequencing analysis, included known genes related to coat color in mammals. The results of real time PCR showed that 8 of the 10 selected genes had significantly higher expression in black sheep skin compared with white sheep skin, which was consistent with the transcriptome sequencing data. Among the differentially expressed genes, *TYR* showed the greatest differential expression between black and white sheep skin (Figure 5).

#### *KE*GG pathway analysis

**Figure 6:**
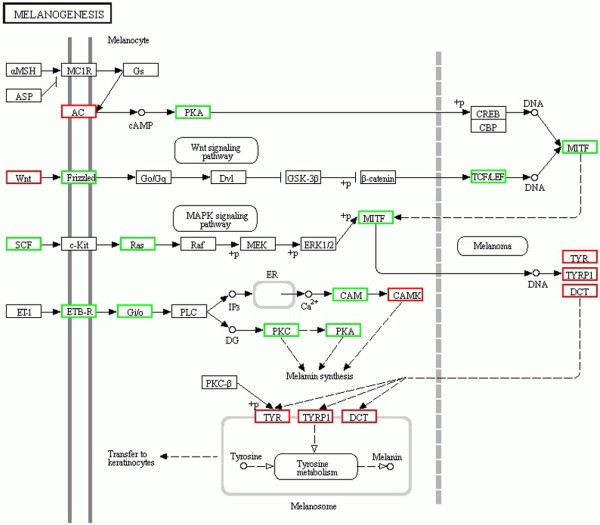
**Differentially expressed coat color genes in sheep skin and their involvement in the melanogenesis pathway.** Genes with red frame are up-regulated and genes with green frame are down-regulated in black versus white sheep skin.

Of the 2,235 known genes differentially expressed in white versus black sheep skin, 1,903 had a specific KEGG pathway annotation. Of these KEGG pathway annotated genes, 324 were down-regulated in black sheep skin. These down-regulated genes are mainly involved in oxidative phosphorylation, and glycolysis and/or gluconeogenesis. Remaining KEGG pathway annotated genes were associated with 241 pathways including those functionally related to coat color in skin such as melanogenesis, tyrosine metabolism and Wnt signaling. For example, there were 20, 53 and 27 differentially expressed genes involved in tyrosine metabolism, Wnt signaling and melanogenesis pathways, respectively. The enriched GO terms for genes identified in sheep skin transcriptome related to pigmentation and melanogenesis and their relative expression in black versus white skin are shown in Table 4 and Figure 6.

**Table 4:** Differentially expressed genes and their GO terms related to pigmentation and melanogenesis in black versus white sheep skin

**GO terms**	**Genes**	**Relative expression in black vs white sheep skin**	**Fold change**	**FDR p-value**	**Corrected p-value of GO term**
Developmental pigmentation	Tyrosinase	Up-regulation	96.88	1.89 × 10^-137^	0.00273
	G-protein coupled receptor 143	Up-regulation	35.63	1.32 × 10^-28^	
	Pallidin-like protein	Down-regulation	0.16	1.47 × 10^-17^	
	P protein-like	Up-regulation	17.65	4.78 × 10^-5^	
	Melanophilin	Up-regulation	27.75	1.94 × 10^-158^	
Pigment cell differentiation	Pallidin-like protein	Down-regulation	0.16	1.47 × 10^-17^	1
	P protein-like	Up-regulation	17.65	4.78 × 10^-5^	
	Melanophilin	Up-regulation	27.75	1.94 × 10^-158^	
Cellular pigmentation and pigment granule localization	G-protein coupled receptor 143	Up-regulation	35.63	1.32 × 10^-28^	1
	Melanophilin	Up-regulation	27.75	1.94 × 10^-158^	
Pigment metabolic process involved in developmental pigmentation and pigmentation	Tyrosinase	Up-regulation	96.88	1.89 × 10^-137^	1
	G-protein coupled receptor 143	Up-regulation	35.63	1.32 × 10^-28^	
	P protein-like	Up-regulation	17.65	4.78 × 10^-5^	
Melanin metabolic process	Aralkylamine N-acetyltransferase (AANAT)	Up-regulation	9.80	7.9 × 10^-5^	1
	Chain E, Crystal Structure Of The 14-3-3 Zeta:serotonin N- Acetyltransferase	Up-regulation	7.84	1.44 × 10^-6^	
	Tyrosinase	Up-regulation	96.88	1.89 × 10^-137^	
	P protein-like	Up-regulation	17.65	4.78 × 10^-5^	
	Membrane-associated transporter protein-like isoform 1	Up-regulation	32.75	1.03 × 10^-42^	
	Membrane-associated transporter protein-like isoform 2	Up-regulation	4099.12	3.46 × 10^-13^	

### Differential expression of known coat color genes

Approximately 127 genes involved in different pathways controlling coat color formation have been identified in the mouse [15]. Those known coat color genes are routinely classified into six general functions including: Melanocyte development, Components of melanosomes and their precursors, Melanosome construction/protein routing, Melanosome transport, Eumelanin and pheomelanin and Systemic effects [15]. Expression of a total of 49 of aforementioned coat color genes was detected in sheep skin in present studies, and 13 genes showed higher expression in black sheep skin and 8 genes showed higher expression in white sheep skin. Interestingly, all genes encoding for the components of melanosomes and their precursors had higher expression in black sheep skin (see Additional file 4: Table S4). The coat color genes in the ‘Eumelanin and pheomelanin’ functional category showed higher expression in black sheep skin, while genes in the ‘Melanosome construction/protein routing (HPS-related)’ category displayed lower expression in black sheep skin. Among the coat color genes showing higher expression in black sheep skin, *TYRP1* showed the highest expression in black sheep skin versus white sheep skin, followed by *TYR*, *MLPH*, *MATP* and *Si* (Table 5). The genes associated with oculocutaneous albinism (OCA) such as *HPS1*, *HPS3*, *HPS4*, *HPS5* and *HPS6* were expressed in sheep skin but most of them did not show differential expression associated with coat color.

**Table 5:** Highly up-regulated coat color genes in black sheep skin

**Gene name**	**Fold change**	**FDR P-value**	**Classification**	**Function**
Membrane-associated transporter protein (*Matp*)	32.75	1.03 × 10^-42^	Components of melanosomes and their precursors	Apparent transporter
Silver (Si)	25.2	0	Components of melanosomes and their precursors	Melanosome matrix
Tyrosinase (*Tyr*)	96.88	1.89 × 10^-137^	Components of melanosomes and their precursors	Melanosomal enzyme
Tyrosinase-related protein 1 (*Tyrp1*)	284.17	0	Components of melanosomes and their precursors	Melanosomal protein
Melanophilin (*Mlph*)	27.75	1.94 × 10^-158^	Melanosome transport	Melanosome transport

## Discussion

Mammalian coat color exhibits a wide range of shades and is dictated by melanin production in melanocytes (melanogenesis). Melanogenesis involves a complex molecular regulation [7]. In order to understand the molecular mechanisms of coat color formation, previous studies have reported the generation of ESTs from both sheep and alpaca skin through traditional Sanger sequencing [16,17]. A previous study examined differences in gene expression associated with black spots in fleece of Corriedale sheep using microarray technology [18]. To further investigate genes that may play important roles in sheep skin, particularly in fiber/coat pigmentation, over 100 million transcriptome sequence reads were generated from white and black sheep skin using the Illumina technology. From these reads there were 37,768 known unigenes identified as expressed in sheep skin, among which 2,235 were differentially expressed in black versus white sheep skin. It is acknowledged that study design was not optimal due to limited biological replication because single pooled samples (n = 3 per coat color) were used in transcriptome sequencing analysis and the same three samples from white sheep skin and from black sheep skin were used individually for quantitative real time PCR validation of the sequencing results. Despite such limitations, results have significantly enhanced understanding of sheep skin transcriptome composition and potential differences in gene expression associated with coat color that are foundational to further study in the future.

Genes encoding for ribosomal proteins, keratin family members and keratin associated proteins were among the most highly expressed genes detected in sheep skin. The ribosome is a central player in the translation system and its function is to decode the nucleotide sequence carried by the mRNA and convert it into an amino acid primary structure [19]. Abundant presence of ribosomal proteins in sheep skin suggests the importance of high rates of protein translation in sheep skin. In channel catfish skin, the expression of ribosomal proteins was high presumably due to higher levels of translational activities [20,21]. Of the top 30 highly expressed genes in sheep skin, all 9 keratin family members and keratin associated proteins displayed down regulation in black sheep skin, which was the same as observed in piebald Merino sheep skin [12]. Collectively, results support Garcia’s view that no single keratin gene alone appears to be responsible for the coat color trait [12]. Hair keratins contain a much higher content of cysteine residues in their non-helical domains and thus form tougher and more durable structures via intermolecular disulfide bond formation [22]. Therefore, high expression of keratins is likely crucial for fleece strength. Genes encoding for important oxidative and dehydrolytic enzymes such as *NADH5* and *COX1* were also highly expressed in sheep skin. The coenzyme NAD (nicotinamide adenine dinucleotide) is a key electron-carrier which mediates hundreds of reactions. The redox state of the NAD–NADH couple plays a central role in energy metabolism [23], signal transduction [24], and transcriptional regulation [25], which is consistent with the need for mitochondrial biogenesis, energy and other proteins during the strong metabolism characteristic of adult sheep skin development [26].

The human hair follicle (HF) has a variable response to potent androgens, such as testosterone (T) and dihydrotestosterone (DHT). The pilosebaceous unit (including HF and sebaceous gland) enzymatically converts weak androgens, such as dehydroepiandrosterone (DHEA) and androstenedione (AD), to more potent androgens, such as T and DHT. In HF of scalp, androgens shorten the anagen growth phase of the hair cycle, causing the HF to regress and recede. The conversion of androgens is dependent on oxidized-reduced pyridine cofactors, NAD, NADH, and NADPH [27]. So, the high level of expression of *NADH* likely improves the conversion of androgens in certain body regions, influencing terminal hair growth.

Transcription factors (TFs) perform important regulatory functions by controlling a variety of cellular processes [28]. In the mouse genome, 1,445 genes were identified to encode for TFs and 983 were expressed in the brain [29]. In the current studies expression of 527 TF genes was detected in sheep skin, including general TFs such as endothelial differentiation-related factor 1 isoform alpha, *DLX3*, *JUN*, *ATF4* and *GATA3*. The high level of expression of these genes detected in skin reflects their importance in regulation of general transcriptional pathways in sheep skin.

Several novel genes were also identified in sheep skin, and a portion of such genes were differentially expressed. Two of the novel genes detected lacked ORF in sequence reads detected, and were highly abundant and exclusively expressed in black sheep skin. BLAST analysis of these 2 novel genes did not find any similar sequences in NCBI database (including EST), suggesting that they could be specific to sheep skin. The differentiated phenotype of melanocytes must be due, at least in part, to differential transcription of melanocyte-specific genes [30]. Thus, these two novel genes may play an important role in promoting pigmentation and dark coat colors.

The GO and KEGG pathway analyses of differentially expressed genes revealed that most were associated with the function of cell and cell part ontology categories. Of particular interest in our dataset were pathways related to pigmentation and melanogenesis. Of the differentially expressed genes, the genes in the category related to ‘the components of melanosomes and their precursor’ and ‘Eumelanin and pheomelanin’ were up-regulated in skin from sheep with black coat color. The function of genes in “the components of melanosomes and their precursor” and ‘Eumelanin and pheomelanin’ are melanin synthesis and the switch between eumelanin and pheomelanin [15]. The darker pigmentation of skin, and possibly of hair, is associated with a higher numbers of melanosomes, although the number of melanocytes remains constant [7,31]. Melanocytes in black hair follicles contain the greatest number of melanosomes (which are eumelanosomes), while the melanosomes in brown hair bulbs are smaller and those in blonde hair are very poorly melanised. The relationship of less melanin with lighter skin/hair phenotype has been reported in several species, including humans [32], alpaca [17], llama [33] and horse [34]. In both domestic sheep and Soay sheep, light coat color is known to be due to a decrease in the ratio of eumelanin to pheomelanin, relative to black coat color [35].

Genes in the ‘Melanosome construction/protein routing (HPS-related)’ ontology category, such as *HPS5*, Lysosomal trafficking regulator (*Lyst*) and *Pallidin*, were all down-regulated in skin of sheep with black coat color. The functions of genes in the ‘Melanosome construction/protein routing (HPS-related)’ categories are related to organelle biogenesis [15]. The key to melanin production is the organelle that is the site of melanogenesis, the melanosome, whose architecture, intracellular distribution and enzyme catalog are critical [30]. HPS5 protein is a component of the biogenesis of lysosome-related organelles complex-2 (*BLOC-2*) and its deficiency can result in Hermansky–Pudlak syndrome (HPS-5) [36]. HPS is a disorder of lysosome-related organelle (melanosome) biogenesis, resulting in oculocutaneous albinism [37,38]. It has been reported that HPS5 melanocytes have an approximately normal contingent of the melanogenic protein, *TYR*[36]. Elucidation of the relationship between lower level of expression of *HPS5* and other genes in this ontology category with black coat color phenotype will require further investigation.

Among the differentially expressed coat color genes, *TYRP1* showed the greatest level of differential expression in black versus white sheep skin. *TYRP1*, one of the members of the tyrosinase family, is a I type membrane bound protein that is expressed in both melanocytes and the retinal epithelium. *TYRP1* is involved in the distal eumelanic pathway and plays a role in stabilizing *TYR*, which is the critical and rate-determining enzyme in melanogenesis [39]. There existed a significant association between coat color and *TYRP1* in Soay sheep [11]. In the free-living Soay sheep, coat color is either dark brown or light tawny color. The light phenotype is determined by homozygosity of a single recessive amino acid change (G → T transversion) at coding position 869 in the *TYRP1* gene [11]. This is consistent with studies in domestic sheep, where light coat color is known to be due to a decrease in the ratio of eumelanin to pheomelanin, relative to black coat color [35].

## Conclusions

In summary, to our knowledge this is the first report of transcriptome analysis of sheep skin from animals with white and black coat color. The present studies have described and revealed a set differentially expressed known and novel genes in sheep skin potentially related to coat color and other physiological functions. The 16 novel genes exclusively expressed in skin of sheep with black coat color are of particular interest for further studies to elucidate their functional roles in coat color regulation. Results are foundational for future studies to potentially manipulate coat color via pharmacological and genetic approaches.

## Methods

### Sheep skin sampling and total RNA extraction

Housing and care of sheep and collection of skin samples for use in the described experiments were conducted in accordance with the International Guiding Principles for Biomedical Research Involving Animals (http://www.cioms.ch/frame 1985 texts of guidelines.htm). The animals were locally anaesthetized with hydrochloridum (1.5 ml of 3%, i.h.), following the approval (reference number 2010[088]) provided by the Animal Hospital of Shanxi Agricultural University to decrease the animal suffering. Six healthy 2-year-old white and black female Sunite sheep 3 sheep per color) were selected for sample collection from the sheep farm in Sunite, Inner Mongolia, China. A piece of skin (8 mm in diameter) from the neck was collected via punch skin biopsy under local anesthesia and immediately placed in liquid nitrogen. Total RNA from the sample was extracted using Trizol reagent (Invitrogen, USA) according to the manufacturer’s instructions. The RNA integrity was evaluated by gel electrophoresis and the RNA purity was checked by the ratio of OD_260_/OD_280_ and RIN value. RNA samples with RIN value greater than 7.5 and OD_260_/OD_280_ ratio greater than 1.7 were selected for deep sequencing.

### Library generation and sequencing

Three RNA samples from black or white sheep skin were pooled before mRNA isolation. Beads with Oligo (dT) were used to isolate poly (A) mRNA from sheep skin total RNA. The isolated mRNA was fragmented followed by first-strand cDNA synthesis using random hexamer-primers. The second-strand cDNA was synthesized using buffer, dNTPs, RNaseH and DNA polymerase I. The short cDNA fragments were purified using QiaQuick PCR extraction kit (Qiagen, USA). The fragment ends were repaired and A tailed followed by ligation to sequencing adaptors. Suitable size fragments were selected following agarose gel electrophoresis and used as templates for PCR amplification. Sequencing of the library was performed using Illumina HiSeq™ 2000.

### Unigene assembly and functional annotation

Raw reads were cleaned by removing adaptors and low quality reads before assembly. Unigene assembly was carried out using the short reads assembly program, Trinity (http://www.genomics.cn). Blastx alignment (e-value < 0.00001) between the unigenes and protein databases (nr, Swiss-Prot, KEGG and COG) was performed, and the best aligned results were used to decide sequence direction of the unigenes. If results of different databases conflicted with each other, a priority order of nr > Swiss-Prot,>KEGG > COG was followed when deciding sequence direction of the unigenes. If a unigene was not aligned to one of the above databases, ESTScan software was used to determine its sequence direction. Unigene sequences were first aligned by blastx to protein databases and then aligned by blastn to nucleotide database nt (e-value < 0.00001), retrieving proteins with the highest sequence similarity with the given unigenes along with their protein functional annotations. Proteins with the highest ranks in the blast results were taken to determine the coding region sequences of unigenes. Coding sequences were translated into amino acid sequences with the standard codon usage. Gene Ontology (GO) functional annotation was based on nr annotation [40]. Blast2GO program (http://www.blast2go.com) was used to assign GO annotations, and WEGO software (http://wego.genomics.org.cn/cgi-bin/wego/index.pl) was used to perform GO functional classification for all unigenes.

### Identification of differentially expressed genes and pathway analysis

A rigorous algorithm based a previously described method [14] was used to identify differentially expressed genes between white and black skin. The FDR (false discovery rate) value of ≤ 0.001 and RPKM ratio of > 2 were used in the analysis [41]. Differentially expressed genes (DEG) were mapped to each term of GO database (http://www.geneontology.org/) and the gene numbers for each GO term were calculated. A list of genes and gene numbers for every GO term was obtained. Hypergeometric test was used to find significantly enriched GO terms in DEG against the genome background. The calculated p-values went through Bonferroni correction, using corrected p-value ≤ 0.05 as a threshold. GO terms fulfilling this condition were defined as significantly enriched GO terms in DEG. With the help of KEGG [42] pathway database (http://www.genome.jp/kegg/pathway.html), the biological complex behaviors of the DEG were further studied.

### Validation of mRNA expressed differentially in skin of sheep with white versus black coat color

Ten genes were selected at random from the differentially expressed genes for validation by quantitative real time PCR analysis. Total RNA was isolated from the same 6 sheep skin samples used for RNA sequencing. One μg of DNase-treated RNA was converted to cDNA using oligo dT primer and MMLV cDNA kit mix (TaKaRa, Dalian, China). The cDNA was then used for real time PCR quantification of mRNAs using mRNA specific primers (Additional file 5: Table S5). β-actin was used as an endogenous control. Quantitative real-time PCR was performed in triplicate on the Stratagene Mx3005P system. The 10 μL PCR reaction included 5 μL SYBR Premix Ex TaqTM II (TaKaRa, Dalian, China), 0.5 μL specific forward primer, 0.5 μL reverse primer, 0.5 μL ROX reference dye, 2 μL diluted (4 times) cDNA and 1.5 μL water. Cycling parameters were 95°C for 30 sec, followed by 40 cycles of 95°C for 5 sec, 56°C or 58°C for 20 sec and 72°C for 15 sec. Melting curve analyses were performed following amplifications. Quantification of selected mRNA transcript abundance was performed using the comparative threshold cycle (CT) method [43]. The difference in abundance of mRNA for the genes was determined by analysis of variance.

## Abbreviations

MITF: Microphthalmia-associated transcription factor; TYRP1: Tyrosinase-related protein 1; TYR: Tyrosinase; DCT: Tyrosinase-related protein2; MLPH: Melanophilin; MATP: Membrane-associated transporter protein; Si: Silver; MC1R: Melanocortin 1 receptor; ASIP: Agouti/agouti signaling protein.

## Competing interests

The authors declare that they have no competing interests.

## Authors’ contributions

RWF designed the study, performed the analysis and wrote the paper. JBY and GWS participated in the design of the study, analyzed the data and critically revised the manuscript. JMB, JSX and XYJ performed real time PCR. LY and YFS took part in sample collection and RNA extraction. RB, HDW, WJG, XYH, MH and XT took part in the data analysis and discussion of the manuscript. CSD conceived the study, analyzed the data and participated in writing. All authors read and approved the final manuscript.

## Supplementary Material

Additional file 1: Table S1 Transcription factors expressed in sheep skin. List of transcription factors and their expression levels in sheep skin. Click here for file

Additional file 2: Table S2 Differentially expressed known genes in black versus white sheep. List of known genes expressed differentially with fold change and P value. Click here for file

Additional file 3: Table S3 Differentially expressed novel genes in black versus white sheep skin. List of novel genes expressed differentially with fold change and P value. Click here for file

Additional file 4: Table S4 Differentially expressed known coat color genes in black versus white sheep skin. List of coat color genes expressed differentially with fold change and P value. Click here for file

Additional file 5: Table S5 Primers used for quantitative real time PCR. List of primers for 10 genes used in quantitative real time PCR analysis to verify differential expression of genes identified by RNA-Seq analysis. Click here for file
