# Supplementary material for: Skin transcriptome profiles associated with coat color in sheep
Source: BMC Genomics. 2013 Jun 10;14:389. doi: 10.1186/1471-2164-14-389 (PMC3689618; doi:10.1186/1471-2164-14-389)
Supplement: Additional file 5: Table S5 — Primers used for quantitative real time PCR. List of primers for 10 genes used in quantitative real time PCR analysis to verify differential expression of genes identified by RNA-Seq analysis. [file 1471-2164-14-389-S5.docx]

**Supplementary Table 5. Primers used for quantitative real time PCR**

| **Gene** | **Primers (5′→3′)** |
| --- | --- |
| P protein like | F: CCCAGACAGGCACCAAGGGC  R: TGGTGGTCTGGGTCTCAGGCA |
| TYRP2 | F: AGGCCGCCTCCACCCCATAG  R: AGGCAGGTTCCTCGCCCAGT |
| EDN3 | F: TTTGCCTCTTCTTCCTTA  R: TATTCTCGGTAGTGTCCC |
| MLPH | F: TCACCTCCTCAGAGCGCCCC  R: GGGGCTGGGTTTGCTCCCTG |
| TYR | F: GCTTTAGCAACTTCATGGGA  R: CTTGTTCTTCTCTGGGACAC |
| MITF | F: TCCCAAGTCAAATGATCCAG  R: GAGCCTGCATTTCAAGTTCC |
| TGF--β1 | F: CACGTGGAGCTGTACCAGAA  R: GAACCCGTTGATGTCCACTT |
| SCF | F: ACCTGGAAGACTTGCTGAGC  R: TGATGTCTCGGGCTAGACCA |
| MATLP1 | F:GGATGGCAGCAGCAGAGGGC  R: CAGTGCCACCGCAGAGGCTG |
| MATLP2 | F: GGATGGCAGCAGCAGAGGGC  R: CCCAGGGCCACGGAGAAGGT |
| β-actin | F: AGCCATGTACGTAGCCATCC  R: ACCCTCATAGATGGGCACAG |

F, forward primer; R, reverse primer
